# Rapid evolution in response to introduced predators II: the contribution of adaptive plasticity

**DOI:** 10.1186/1471-2148-7-21

**Published:** 2007-02-14

**Authors:** Leigh C Latta, Jeremy W Bakelar, Roland A Knapp, Michael E Pfrender

**Affiliations:** 1 Department of Biology, 5305 Old Main Hill Road, Utah State University, Logan, UT 84322, USA; 2 Sierra Nevada Aquatic Research Laboratory, University of California, HCR 79, Box 198, Mammoth Lakes, CA 93546, USA

## Abstract

**Background:**

Introductions of non-native species can significantly alter the selective environment for populations of native species, which can respond through phenotypic plasticity or genetic adaptation. We examined phenotypic and genetic responses of *Daphnia *populations to recent introductions of non-native fish to assess the relative roles of phenotypic plasticity versus genetic change in causing the observed patterns. The *Daphnia *community in alpine lakes throughout the Sierra Nevada of California (USA) is ideally suited for investigation of rapid adaptive evolution because there are multiple lakes with and without introduced fish predators. We conducted common-garden experiments involving presence or absence of chemical cues produced by fish and measured morphological and life-history traits in *Daphnia melanica *populations collected from lakes with contrasting fish stocking histories. The experiment allowed us to assess the  degree of population differentiation due to fish predation and examine  the contribution of adaptive plasticity in the response to predator  introduction.

**Results:**

Our results show reductions in egg number and body size of *D. melanica *in response to introduced fish. These phenotypic changes have a genetic basis but are partly due to a direct response to chemical cues from fish via adaptive phenotypic plasticity. Body size showed the largest phenotypic change, on the order of nine phenotypic standard deviations, with approximately 11% of the change explained by adaptive plasticity. Both evolutionary and plastic changes in body size and egg number occurred but no changes in the timing of reproduction were observed.

**Conclusion:**

Native *Daphnia *populations exposed to chemical cues produced by salmonid fish predators display adaptive plasticity for body size and fecundity. The magnitude of adaptive plasticity was insufficient to explain the total phenotypic change, so the realized change in phenotypic means in populations exposed to introduced fish may be the result of a combination of initial plasticity and subsequent genetic adaptation. Our results suggest that immediately following the introduction of fish predators, adaptive plasticity may reduce the impact of selection through "Baldwin/Bogert effects" by facilitating the movement of populations toward new fitness optima. Our study of the response of a native species to an introduced predator enhances our understanding of the conditions necessary for rapid adaptive evolution and the relationship between rapid evolution and adaptive phenotypic plasticity.

## Background

Introductions of non-native species can result in strong selective challenges for native populations. The strength of selection in this case is determined by the size of the environmental shift, which imposes a fitness cost on the population proportional to the squared distance between the population mean phenotype and the position of the new optimum [1,2]. If the optimum moves far enough, the fitness cost will be sufficiently high to reduce the intrinsic rate of increase of the population to <1. Unless the population can rapidly advance toward the new optimum phenotype, it will not persist [3]. Two processes can facilitate persistence of populations challenged with a rapidly changing environment: adaptive phenotypic plasticity and genetic adaptation.

Adaptive phenotypic plasticity allows individuals within a population to accommodate a changing environment [4,5] by facilitating rapid movement to a new fitness optimum. This movement occurs through changes in the mean value of a trait and/or changes in the genetic and phenotypic variance/covariance structures. In the extreme case, plastic changes in the mean value of a trait are able to completely move a population to a new fitness optimum and no genetic adaptation is required [5]. In cases where a plastic change in the mean is not sufficient to shift a population to a new optimum it can allow a population to persist until sufficient adaptive genetic changes occur [6,7]. An incomplete shift in the population mean towards a new selective optimum that facilitates population persistence is called the "Baldwin effect" [6] and the resulting reduction in the intensity of selection is referred to as the "Bogert effect" [8] or adaptive buffering [9]. Plastic changes in the (co)variance matrix may result in increased levels of expressed genetic variance (*i.e*., variance that is context-dependent and arises only in response to specific environmental cues) and changes in covariances between traits that increase the response to selection [10-13].

Populations may also adapt genetically to new environmental conditions when there is no pre-existing adaptive phenotypic plasticity or plasticity is insufficient to completely shift a population to a new phenotypic optimum. The rate of genetic adaptation toward a new optimum is determined by a number of factors, including the amount of additive genetic variation present for the traits under selection [14], the rate at which mutation produces new adaptive variation [15,16], and genetic correlations among characters [16-19].

A common source of rapid environmental change arises from the introduction of novel predator species. In a notable example, non-native fishes have been widely introduced into naturally fishless alpine lakes throughout the world and have had profound effects on native zooplankton species, including *Daphnia*. *Daphnia *have a long history as a model system to study the consequences of introduced fish predators [20-23]. *Daphnia *adapt to introduced fish through changes in traits related to detection avoidance, including alterations in patterns of diel vertical migration (DVM) [24,25] and reduced body size [21,23,26]. *Daphnia *also display significant adaptive phenotypic plasticity in response to chemical cues produced by fish that can facilitate persistence during changes in selection regime. Plastic changes that reduce pigmentation [27] and body size [28-32] in *Daphnia *decrease the ability of fish to detect their prey resulting in higher survivorship, while plastic increases in fecundity [28-30] result in higher intrinsic rates of population increase.

*Daphnia melanica *(identified as *Daphnia middendorfiana *in previously published studies, *e.g*., [33-35], but recently classified as *D. melanica *based on molecular analyses [M. Pfrender, unpublished data]) populations located in alpine lakes throughout the Sierra Nevada in eastern California, USA provide a unique opportunity to study the effects of introduced predators on naive populations. These alpine lakes have been the subjects of extensive ecological study [33-35] in part because the history of fish introductions is well documented. In lakes where *D. melanica *and fish co-occur, *D. melanica *have smaller body sizes and reproduce earlier relative to those in lakes without fish [36]. These differences were attributed to rapid adaptive evolution. However, because *Daphnia *are often highly plastic in response to chemical cues from fish, the differences in morphology and life-histories observed previously may not be solely due to underlying genetic alteration. Differences in morphology and life-history could be entirely due to phenotypic plasticity or a combination of plastic and genetic modification.

To determine the relative contributions of adaptive plasticity and genetic adaptation during rapid evolution in response to introduced fish we conducted common-garden experiments on clonally reproducing females of *D. melanica *populations collected from four lakes in the Sierra Nevada with contrasting fish stocking histories. Two lakes were never stocked and remain in their natural fishless condition and two lakes have contained introduced fish populations during the last several decades. We measured morphological and life-history traits of clonally reproducing females from each population in the presence and absence of chemical cues from fish (*i.e*., fish kairomone). Because *D. melanica *can be maintained in a state of constant clonal reproduction in the lab, it is straightforward to utilize standard quantitative genetic techniques to estimate the contribution of genetic and plastic phenotypic effects underlying adaptive traits. Our chief working assumption in this experiment is that the phenotypic states of fishless populations are representative of the ancestral phenotypic states of populations that currently contain fish. Given our assumption of equality between currently fishless populations and ancestral states of fish populations is true, our study design allowed us to determine the degree of morphological and life-history differentiation due to selection by fish predation and quantify the contribution of phenotypic plasticity in determining adaptive responses to the introduction of fish.

## Results

### Number of eggs and fecundity

Due to occasional mortality in the life-table prior to release of first clutch we measured egg number as an index calibration for fecundity to increase our sample sizes. For individuals that had both egg number and number of live offspring measured egg number was a highly significant predictor of the number of viable offspring (all regressions: p < 0.01). All regressions (described in more detail in the methods section) showed a positive correlation between the two variables. Correlation coefficients for the data subsets were between 0.43 and 0.57.

### Levels of phenotypic plasticity

**Figure 1:**
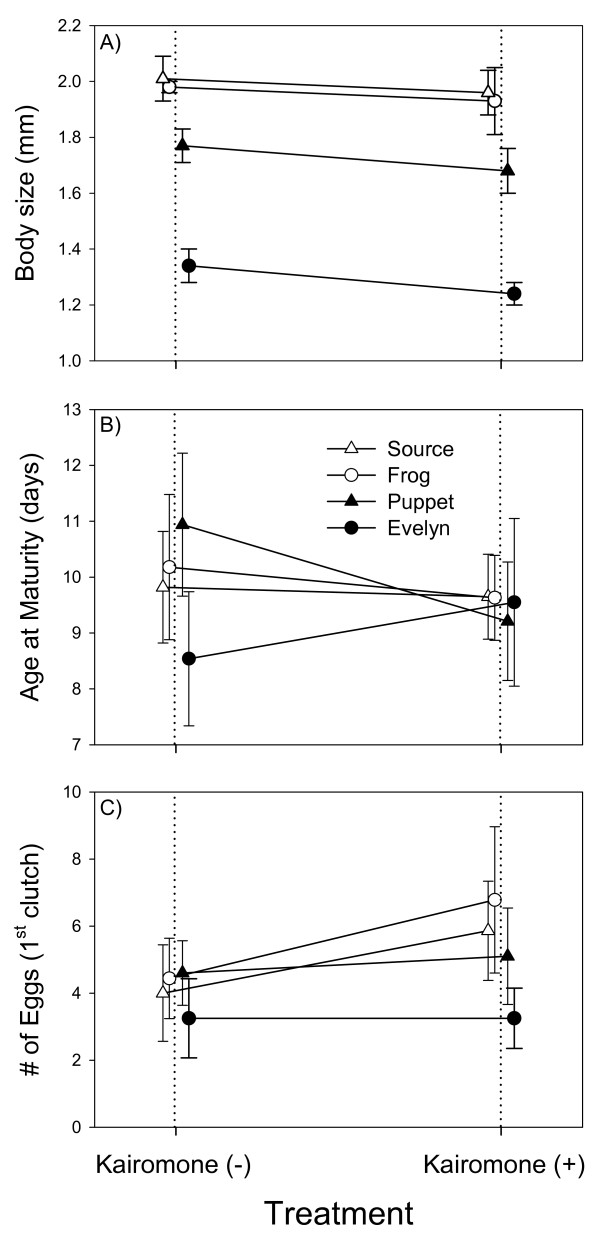
**Population responses to fish kairomone**. Reaction norm plots for a) body size, b) age at maturity, and c) number of eggs in response to presence (+) or absence (-) of fish kairomone. Open symbols left of the vertical dotted lines are values for fishless populations, filled symbols right of the vertical dotted lines are values for fish-containing populations. Error bars are +/- one standard error of the mean.

Fish kairomone caused significant reductions in mean body size at maturity for all populations (Fig. 1A). In the kairomone(-) treatment, mean body size at maturity for all genotypes was 1.78 mm, while the average size at maturity in the kairomone(+) treatment was 1.70 mm. Non-significant interaction terms suggest that a population's response to fish kairomone is independent of its history of fish introductions. Mean age at maturity did not change in response to fish kairomone (Fig 1B). This result appears largely as a consequence of the large variances associated with this trait. The number of eggs in the brood pouch increased in response to fish kairomone (Fig. 1C). The number of eggs increased significantly from 4.1 in kairomone(-) to 5.2 in kairomone(+) in response to fish kairomone (Table 1). Although there is a tendency for fishless populations to produce more eggs in response to kairomones than fish populations, the difference in reaction norms between fishless and fish populations is not significant so changes in clutch size are also independent of the history of fish introductions.

**Table 1:** NANOVA results.

	**Trait**								
	**Size**			**Age**			**Egg #**		

**Effect**	**df**	**F**	**p**	**df**	**F**	**p**	**df**	**F**	**p**

Environment	1/60	6.87	**0.0111**	1/60	0.81	0.3726	1/60	5.41	**0.0235**
Type	1/60	278.56	**<0.0001**	1/60	0.42	0.5196	1/60	5.85	**0.0186**
Pop(Type)	2/60	66.06	**<0.0001**	2/60	1.79	0.1760	2/60	3.11	0.0519
Environment × Pop(Type)	2/60	0.02	0.9776	2/60	3.13	0.0507	2/60	0.12	0.8890
Environment × Type	1/60	0.71	0.4014	1/60	0.00	0.9945	1/60	3.35	0.0723

Results from NANOVA for morphological (size) and life-history (age and egg number) traits. Shown are the degrees of freedom (df), F-values (F) and p-values (p). Significant results (p < 0.05) are indicated in bold.

Because levels of variation for a trait are often context dependent we calculated coefficients of variation for body size, age at maturity, and egg number to determine if the amount of variance in these traits is dependent upon the presence/absence of fish kairomone. Coefficients of variation were lowest for body size at maturity (range 1.5 – 9.8), intermediate for age at maturity (range 10.5 – 22.1), and highest for egg number (range 32.8 – 51.4). Expressed variance showed little response to fish kairomone based on our criterion of non-overlapping confidence intervals. Variance in body size increased significantly only in the Frog Lake population in the presence of fish kairomone (Table 2).

**Table 2:** Means and CV's.

				**Treatment**			
				**Kairomone (-)**		**Kairomone (+)**	

**Population**	**N**	**Exposure Time**	**Trait**	**Mean (SE)**	**CV (95% CI)**	**Mean (SE)**	**CV (95% CI)**

*Source*	7	0	*Size*	2.01 (0.04)	5.0 (3.2–10.9)	1.96 (0.04)	5.1 (3.3–11.3)
			*Age*	9.82 (0.50)	13.4 (8.6–30.3)	9.65 (0.38)	10.5 (6.7–23.4)
			*Egg #*	4.00 (0.72)	47.8 (28.9–165.2)	5.86 (0.74)	33.3 (20.8–86.9)
*Frog*	9	0	*Size*	1.98 (0.01)	1.5 (1.0–2.9)	1.93 (0.06)	9.8 (6.6–19.0)
			*Age*	10.18 (0.65)	19.2 (12.8–38.1)	9.63 (0.38)	11.7 (7.9–22.8)
			*Egg #*	4.44 (0.60)	40.8 (26.3–95.2)	6.78 (1.09)	48.2 (30.7–125.4)
*Puppet*	10	53	*Size*	1.77 (0.03)	6.2 (4.3–11.4)	1.68 (0.04)	7.7 (5.3–14.2)
			*Age*	10.94 (0.64)	18.6 (12.7–34.5)	9.21 (0.53)	18.2 (12.4–34.3)
			*Egg #*	4.60 (0.48)	32.8 (22.0–66.6)	5.10 (0.72)	44.7 (29.3–101.5)
*Evelyn*	8	91	*Size*	1.34 (0.03)	6.0 (3.9–12.2)	1.24 (0.02)	5.6 (3.7–11.5)
			*Age*	8.54 (0.46)	15.2 (10.0–31.8)	9.55 (0.75)	22.1 (21.5–47.6)
			*Egg #*	3.25 (0.59)	51.4 (31.8–163.2)	3.25 (0.45)	39.4 (25.0–99.1)

Estimates of phenotypic means and coefficients of variation. The units for body sizes are mm and for ages at maturity are days. Standard errors for the mean and 95% Modified McKay confidence intervals for coefficients of variation are given in parentheses.

### Genetic differentiation among populations

Genotypes from fishless populations had significantly larger body sizes at maturity than did genotypes from populations that co-exist with fish (Table 1). The average body size of genotypes from fishless lakes was 1.97 mm while genotypes from fish-containing lakes averaged 1.51 mm in size. Post hoc pairwise comparisons based on *t *values between all populations showed that genotypes from fishless populations ("Source" and "Frog") did not differ from one another (p = 0.47), but are significantly larger than genotypes from Puppet Lake (p < 0.0001) and Evelyn Lake (p < 0.0001). Puppet Lake genotypes are also significantly larger than genotypes from Evelyn Lake (p < 0.0001). A non-significant interaction term implies that these differences are not sensitive to the presence of fish kairomone (Table 1).

There was a significant reduction in the number of eggs in the brood pouch, from 5.27 eggs per individual in fishless populations to 4.05 eggs per individual in populations co-occurring with fish (Table 1). However, this result appears largely influenced by one population. Post hoc comparisons show that Evelyn Lake genotypes produce significantly fewer eggs than genotypes from Frog, Source, and Puppet Lake (p = 0.0015, 0.0298, and 0.0241, respectively), but Frog, Source, and Puppet Lakes do not differ in egg production (all possible pairs: p > 0.2602). A non-significant interaction suggests the difference in egg production between Evelyn Lake genotypes and all others did not depend on the assay environment. Age at maturity did not differ among fish and fishless populations (Table 1).

## Discussion

Rapid evolution is an important component of the success of invading species [37] and the response of organisms in invaded communities [9] because it ameliorates the selective cost imposed by a shift in the phenotypic optimum and enhances the probability of long-term population persistence. Similarly, adaptive plasticity may be an important component of rapid evolution as it can allow short-term population persistence following changes in the selective environment [6,8] that in turn provides time for evolutionary mechanisms to operate. However, disentangling actual cases of rapid evolution from purely plastic changes in response to a changing environment can be problematic because adaptive phenotypic plasticity is a common feature in many organisms [38].

In this study we examined the rapid changes of *Daphnia *morphology and life histories in response to a single abrupt change in the environment. Specifically, we investigated the relative role of genetic and plastic phenotypic changes in moving populations exposed to a novel predator toward a new fitness optimum. Our results show that reductions in egg number and body size of *D. melanica *genotypes from the Sierra Nevada, in response to introduced fish, are largely adaptive evolutionary responses and not due entirely to adaptive phenotypic plasticity. We do find evidence for adaptive plasticity, in the form of increases in clutch size and reductions in body size, in these populations that could facilitate short-term persistence and subsequent rapid evolution. We caution, however, that our interpretation of these results is predicated on the assumption that our measured phenotypes of currently fishless populations are representative of the ancestral phenotypes of populations that currently co-exist with fish.

Our results suggest that naive *D. melanica *populations in the Sierra Nevada may initially respond to fish introductions through adaptive phenotypic plasticity brought about by chemical cues from fish, which facilitates movement towards the new phenotypic optimum. First, plastic reductions in body size make *D. melanica *less visible to fish and constitute evidence for a "Baldwin/Bogert effect" [6,8]. Fish are highly effective size-selective predators and their efficiency is primarily linked to prey visibility [26,39-41]. Thus, *D. melanica *that are less visible have a fitness advantage (via increased survivorship) through movement towards the new phenotypic optimum and the resulting reduction in selection intensity due to decreased predator efficiency. Concomitant with a decrease in body size, *D. melanica *also show adaptive phenotypic increases in fecundity that could lead to higher intrinsic rates of population increase. Although the rate at which fish remove individuals from these *Daphnia *populations is unknown, our observed increase in clutch size of approximately one is quite significant. Estimates of *D. melanica *population sizes in the Sierra Nevada are on the order of hundreds of millions to billions [R. Knapp, unpublished data], thus, an increase of one individual at first reproduction might substantially offset any losses due to predation. An interesting aspect of our findings is that our naive *Daphnia *populations, those without any history of fish exposure, are responsive to chemicals produced by fish. This observation suggests that *D. melanica *may be pre-adapted to fish predation, and that the genetic machinery responsible for adaptive phenotypic plasticity in response to fish kairomone is ancestral in this species.

Although we find evidence for adaptive phenotypic plasticity that would facilitate short-term population persistence in the face of novel predation, it is not sufficient to explain the difference in body-size and egg number between populations that are historically fishless and those that co-occur with fish. For example, a comparison of the average body size in the Evelyn Lake population (1.34 mm in the kairomone(-) and 1.24 mm in the kairomone(+) treatments) with the average in the fishless populations (2.00 mm in the kairomone(-) and 1.95 mm in the kairomone(+) treatments) shows that the mean phenotype in Evelyn Lake has diverged by 9.4 phenotypic standard deviations. The change in body size attributable to plasticity in fishless populations is approximately one standard deviation. In other words, the change in body size due to plasticity accounts for only about 11% of the total difference observed between Evelyn and fishless populations. Thus, the phenotypic differences observed in our study appear largely due to changes in the underlying genetic components controlling phenotype.

Our observation that the body-size response in Evelyn Lake was much higher than that in Puppet Lake could arise for three reasons. First, the difference in body size could simply reflect the different amounts of time each population was exposed to fish predation (Puppet Lake – 53 years; Evelyn Lake – 91 years). Second, Daphnia populations may have experienced differing levels of fish predation resulting in varying selection intensities, with the selection intensity in Evelyn Lake substantially higher. Finally, given our observation that expressed levels of genetic variance for body size increased approximately 6-fold in response to fish kairomone in one fishless population (Frog Lake) but not in the other (Source Lake) our fish populations may have differed in the initial levels of standing genetic variation, with the Evelyn Lake population harboring more standing genetic variation than Puppet Lake.

Our observation that Evelyn Lake was the only population to display a significant evolutionary reduction in egg number is likely due to the ability of *D. melanica *to deposit large amounts of melanin in the carapace. Melanin production in the carapace would initially "blind" selection to changes in egg number. Thus, an evolutionary response in egg number should occur only after reductions in melanin deposition. Fish predation produces strong selection on melanin production in other *Daphnia *populations [42], and there is evidence for reduced melanin expression in *Daphnia *from our fish populations relative to fishless populations [M. Pfrender, unpublished data]. Therefore, the apparent delayed onset of selection on egg number could be due to initial selection on melanin production and subsequent selection on egg number.

Traditional views of character evolution typically involve trade-offs among traits that can limit the adaptive potential of a population [43]. However, several selection experiments involving *Daphnia *suggest adaptive evolutionary changes in one trait are not necessarily associated with concomitant maladaptive changes in others [44-46]. We observe a similar result here, where evolutionary and plastic changes in body size and fecundity occur in the absence of changes in the timing of maturity and reproduction. Our results, and those of other researchers that imply the absence of a trade-off, could be attributed to assay conditions in which food is not a limiting resource [47]. *Daphnia *morphology and life-history can display food concentration-dependent reactions to the presence of fish kairomone [48].

## Conclusion

We investigated the relative contributions of selection and adaptive phenotypic plasticity to the rapid evolution of morphology and life histories in response to an introduced predator. We conclude that adaptive plasticity could facilitate short-term population persistence through "Baldwin/Bogert effects", but that long-term persistence was achieved through subsequent genetic adaptation. Further investigation into other traits that may have also undergone rapid change in selective regime as a consequence of fish introductions, such as pigmentation and DVM behavior, examined under differing kairomone and food conditions, will provide a more detailed view of the traits and processes involved in the overall evolution of the *Daphnia*/fish predator-prey system in the Sierra Nevada.

Numerous studies have examined the contributions of plasticity and selection to rapid adaptation in non-native species following their introduction into a novel environment (*e.g*., [49]). In contrast, few studies have examined the phenotypic and evolutionary response of native species to introduced species that pose strong novel selective challenges. Thus, this study and a growing body of others investigating the response of native communities to introduced species should enhance our understanding of the conditions necessary for rapid adaptive evolution and the relationship between rapid evolution and population persistence [9].

## Methods

### Study populations

Individual genotypes used in the life-table assay were collected from four permanent lakes in the central Sierra Nevada during the summer of 2004. These lakes are located in the Humphreys, French Canyon, and Vogelsang basins at elevations ranging from 3150–3632 meters. Frog Lake (ID# 52103; UTM Zone 11: 351079 E, 4124432 N) and Source Lake (UTM Zone 11; 349988 E 4125708 N), remain in their natural fishless condition (referred to collectively as fishless populations). Puppet and Evelyn Lakes were naturally fishless but were stocked with trout during the past century. Puppet Lake has been stocked with golden trout (*Oncorhynchus mykiss aguabonita*) every other year since 1951 (California Dept. of Fish and Game, unpublished stocking records), resulting in 53 years of fish predation on the resident *D. melanica *population at the time of collection. Evelyn Lake (UTM Zone 11; 295393 E, 4186659 N) was initially stocked with brown trout (*Salmo trutta*) in 1913. Brook trout (*Salvelinus fontinalis*) were introduced in 1928, 1946, 1947, 1949, 1951, 1954 and 1958, and rainbow trout (*Oncorynchus mykiss*) were introduced in 1939, 1942, 1944, 1957, 1962, and 1966 [50]. No stocking has occurred since 1966, and the resident rainbow trout population is self-sustaining. In total, *Daphnia *in Evelyn Lake were exposed to 91 years of fish predation at the time of collection (Puppet Lake and Evelyn Lake are referred to collectively as fish populations).

### Clone establishment and maintenance

*Daphnia *were collected from each of the study lakes and maintained at 4°C for a period of 1–2 weeks prior to isolation in the lab. To capture the maximum amount of genetic variation from each population, mature females from the original field collection were isolated and placed singly in 250 mL beakers containing 200 mL of filtered well-water. This procedure ensures that no isolates were genotypically identical juveniles produced in the period from collection in the field until isolation in the lab. Isolated individuals were maintained by clonal reproduction under constant conditions of temperature (15°C) and 16L:8D photoperiod for approximately 20 generations prior to experimentation. Water levels in the beakers were kept constant with the periodic addition of double-distilled water. *Daphnia *were fed a vitamin supplemented pure culture of the green alga *Scenedesmus obliquus *every 3–4 days.

### Life-table assay

Morphological and life-history characteristics were assayed using a standard experimental design [51,52]. Briefly, single immature females were taken from the stock isolates, each representing an experimental line. The lines were then maintained as single asexually produced progeny for two generations. In third generation individuals, we measured a suite of traits upon reaching maturity (defined as the first instar with the deposition of eggs into the brood pouch). Two traits, number of eggs in the brood pouch and size at maturity are directly related to visibility and potential for survival in the face of visually-feeding predators. The two remaining traits, age at maturity and number of viable offspring produced are related to the intrinsic rate of population increase. Each experimental line was maintained in a 250 mL beaker containing 150 mL of filtered well-water supplemented with a constant concentration (135,000 cells/mL) of *S. obliquus*. Upon reaching maturity, second generation lines assigned to the fish kairomone treatment were placed in filtered well-water aged with a 20–25 cm bull trout (*Salvelinus confluentus*) for 24 hours. (the kairomone treatment is referred to as kairomone(+) and the non-kairomone treatment as kairomone(-)) Exposing second generation individuals to fish kairomone post-maturity ensures that maternal effects due to fish kairomone are minimized. All beakers in the life-table assay were maintained in a controlled temperature room with a 16L:8D photoperiod at 18°C and their position in the chamber changed every two days to minimize micro-environmental differences. The food/water mixture in all beakers was replaced with food/water of the appropriate type, kairomone(+) or kairomone(-), every other day.

### Statistical procedures

We performed linear regression on egg number and number of surviving offspring upon release of first clutch to determine if egg number serves as a proxy for the more general fitness character of fecundity. Regressions were run on four separate subsets of the data: 1) fishless populations in the kairomone(-) treatment; 2) fishless populations in the kairomone(+) treatment; 3) fish populations in kairomone(-) treatment; and 4) fish populations in the kairomone(+) treatment. Analyzing the subsets separately aided in determining whether a correlation between egg number and viable offspring is sensitive to environmental and/or genetic differences between populations.

Nested analysis of variance (NANOVA) in which covariance parameters were estimated using restricted maximum likelihood was performed on three traits (body size at maturity, age at maturity, and egg number at maturity) to test for fixed effects of treatment (kairomone(+) or kairomone(-)), lake type (fish or fishless), population nested within lake type, and interactions between environment and lake type, and between environment and population nested within lake type [PROC MIXED; [53]]. The model was designed to account for heterogeneity in covariance matrices across treatments because variance and covariance estimates can vary across environments.

Interpretation of results based on our model is relatively straightforward. A significant treatment effect is evidence for phenotypic plasticity in a given trait, irrespective of a populations' fish stocking history. A significant lake type effect implies phenotypic differences between populations in historically fishless and fish-containing lakes. The strength of conclusions about the actual level of genetic differentiation underlying phenotypic divergence is based on the level of significance of the interaction term. For example, a significant effect of lake type in conjunction with non-significant interaction terms would indicate underlying genetic differences among populations regardless of treatment effects.

Plasticity in the expressed genetic variance of traits was assessed by calculating coefficients of variation (CV) for each population across treatments separately. We then constructed 95% Modified McKay confidence intervals for each CV [54,55] and assessed differences between estimates based on the degree of overlap of confidence intervals.

## Authors' contributions

LCL designed and conducted the experiment, performed statistical analyses, and drafted the manuscript. JWB conducted the experiment. RAK provided background information on *Daphnia *populations, assisted with fieldwork in the Sierra Nevada, and helped draft the manuscript. MEP conceived of the study, conducted fieldwork in the Sierra Nevada to collect the experimental populations, and contributed to analysis and manuscript preparation. All authors read and approved the final manuscript.
